# Clinical Results of Hypomethylating Agents in AML Treatment

**DOI:** 10.3390/jcm4010001

**Published:** 2014-12-25

**Authors:** Marjan Cruijsen, Michael Lübbert, Pierre Wijermans, Gerwin Huls

**Affiliations:** 1 Department of Hematology, Radboud University Medical Center, PO Box 9191, 6500 HB Nijmegen, The Netherlands; E-Mail: marjan.cruijsen@radboudumc.nl; 2 Department of Medicine, University of Freiburg Medical Center, D-79106, Freiburg, Germany; E-Mail: michael.luebbert@uniklinik-freiburg.de; 3 Department of Hematology, Haga Hospital, 2545 CH, The Hague, The Netherlands; E-Mail: p.wijermans@hagaziekenhuis.nl

**Keywords:** AML, azacitidine, decitabine, hypomethylating agents, elderly, epigenetics

## Abstract

Epigenetic changes play an important role in the development of acute myeloid leukemia (AML). Unlike gene mutations, epigenetic changes are potentially reversible, which makes them attractive for therapeutic intervention. Agents that affect epigenetics are the DNA methyltransferase inhibitors, azacitidine and decitabine. Because of their relatively mild side effects, azacitidine and decitabine are particularly feasible for the treatment of older patients and patients with co-morbidities. Both drugs have remarkable activity against AML blasts with unfavorable cytogenetic characteristics. Recent phase 3 trials have shown the superiority of azacitidine and decitabine compared with conventional care for older AML patients (not eligible for intensive treatment). Results of treatment with modifications of the standard azacitidine (seven days 75 mg/m^2^ SC; every four weeks) and decitabine (five days 20 mg/m^2^ IV; every four weeks) schedules have been reported. Particularly, the results of the 10-day decitabine schedule are promising, revealing complete remission (CR) rates around 45% (CR + CRi (*i.e.*, CR with incomplete blood count recovery) around 64%) almost comparable with intensive chemotherapy. Application of hypomethylating agents to control AML at the cost of minimal toxicity is a very promising strategy to “bridge” older patients with co-morbidities to the potential curative treatment of allogeneic hematopoietic cell transplantation. In this article, we discuss the role of DNA methyltransferase inhibitors in AML.

## 1. Introduction

Epigenetic changes include, by definition, through mitosis and meiosis, heritable changes in gene expression that are not caused by changes in the primary DNA sequence [1]. Epigenetic changes affect the spatial structure of the DNA that is coiled around histones. The spatial structure determines whether the transcription machinery, which transcribes DNA into RNA, can or cannot bind to the promoter of a gene, in order to initiate transcription. The best-known epigenetic changes are methylation and acetylation of amino acid residues in histones and methylation of cytosine (C) bases in areas of the genome rich in CpG dinucleotides (CpG islands). Methylation of cytosines, mediated by one of the DNA methyltransferases (DNMTs), results in silencing of gene expression. DNMT1 maintains existing methylation patterns following DNA replication, whereas DNMT3A and 3B methylate unmethylated CpGs (*de novo* methylation). It is increasingly clear that epigenetic changes play a role in oncogenesis. Cancer cells generally exhibit genome-wide hypomethylation, resulting in genetic instability, and CpG islands hypermethylation, modifying gene expression (e.g., preventing the expression of tumor suppressor genes) [2]. In contrast to genetic changes, epigenetic changes are considered to be reversible. This makes epigenetic changes an attractive candidate for therapeutic intervention. There is considerable evidence that abnormal methylation plays an important role in the pathogenesis of hematological malignancies, including acute myeloid leukemia (AML). AML blasts have distinct methylation patterns compared with normal CD34^+^ cells, and various subtypes of AML, e.g., with mutated NPM1, have distinct methylation profiles [3,4]. Recent studies using massively parallel sequencing technologies have identified mutations of *DNMT3A*, *TET2* and *IDH* (1 and 2) in 12%–22%, 7%–23% and 15%–33%, respectively, of the AML patients [5,6]. These genes are involved in DNA methylation, and therefore, their mutated variants may help elucidate the mechanisms of aberrant DNA methylation in AML blasts. Furthermore, translocations (e.g., *MLL* (mixed lineage leukemia gene)) and mutations (e.g., *ASXL1*, *UTX*) in genes affecting histone modifications are frequently observed.

## 2. DNA Hypomethylating Agents

**Figure 1:**
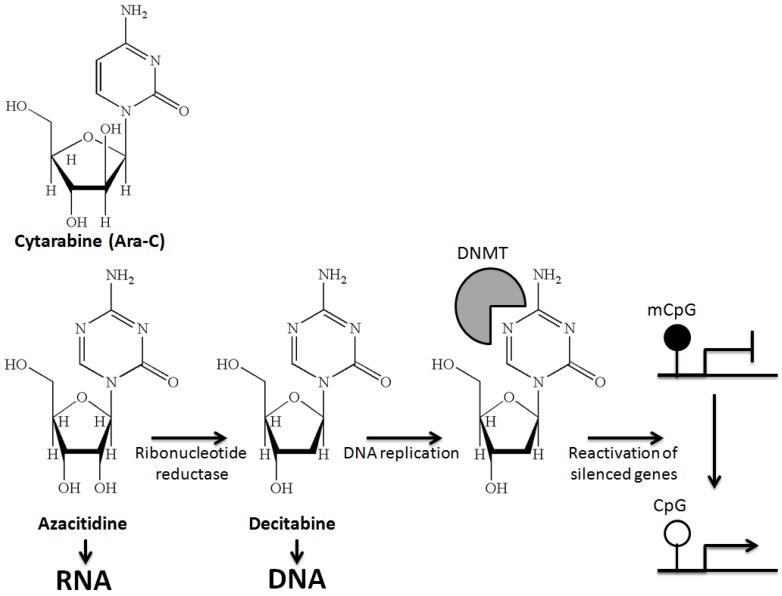
Mechanism of action of hypomethylating agents. (Black circles, methylated CpG; white circles, unmethylated CpG.)

In the 1960s, 5-azacytidine (further called azacitidine) and 5-aza-2′-deoxycytidine (further called decitabine) were synthesized to develop analogs of cytosine (like cytarabine) for the treatment of AML. Although these drugs clearly had anti-neoplastic activity, they turned out to be extremely toxic at high doses. Renewed interest in azacitidine and decitabine arose after discovering the hypomethylating properties of these drugs. The DNA hypomethylating property of azacitidine and decitabine was traced to their ability to incorporate into DNA, trap DNA methyltransferases (DNMTs) and target these enzymes for degradation [7]. DNA synthesis in the absence of these enzymes then results in hypomethylation in the daughter cells and eventually to reactivation of silenced gene expression (Figure 1). It is important to recognize that while inhibiting DNA methylation is a molecularly precise, targeted therapy approach, the downstream effects on neoplastic behavior are quite nonspecific. The trapping of DNMTs onto DNA creates bulky adducts that can inhibit DNA synthesis and eventually result in cell death by cytotoxicity [2,7]. Furthermore, if one considers reactivated genes, these drugs affect multiple pathways, including cell cycle arrest (for example, via p15 activation), apoptosis, differentiation, stem cell renewal, invasion, angiogenesis, immune recognition *etc*. In particular, the pre-clinical data, which suggest that azanucleotides increase the immunogenicity of AML blasts by promoting the expression of silenced antigens (e.g., of melanoma-associated antigens (MAGE)), could become a fruitful lead for future (transplantation) studies [8].

The observation that hypomethylating agents (HMAs) have to be incorporated into DNA to inhibit DNA methylation (and consequently, transcription activation) implies that these agents should be used differently than conventional chemotherapy. HMAs have to be given for at least 3–6 cycles before it can be concluded whether they have activity against the disease (or not). These drugs should be given at a standard dose at fixed times, despite the presence of cytopenias. Further, these drugs can have meaningful clinical activity (e.g., transfusion independency) and improve survival, despite the fact that no CR is achieved. Indeed, the recovery of peripheral blood counts and quality of life are important reasons to continue treatment with HMAs.

## 3. Azacitidine and AML

Azacitidine was tested in two separate phase 3 studies in myelodysplastic syndromes (MDS) [9,10]. Response rates ranging from 30% to 60% were observed, with documented improved survival compared with either supportive care or cytotoxic chemotherapy. Since some of the included patients would currently be considered as AML patients (according to WHO criteria), this allowed studying the anti-leukemic effect of azacitidine in AML patients. In the Cancer and Leukemia Group B (CALGB) 9221 study, 27 patients with AML were randomly assigned to azacitidine and 12 AML patients were assigned to observation [11]. The median survival time for the treated patients was 19.3 months compared to 12.9 months in the group assigned to observation (*n =* 25; including the control arms of CALGB 8421 and 8921). A post hoc analysis of the pivotal MDS001 trial for older patients who met the WHO criteria for AML (*i.e.*, 20%–30% bone marrow (BM) blasts) showed 18% CR, with a survival benefit in favor of azacitidine (24.5 *vs.* 16 months, *p =* 0.005), including higher two-year OS (38% *vs.* 0%, *p =* 0.01) in patients with adverse cytogenetics [12].

Recently, the data of the AML001 study, a global, multi-center, randomized study, including 488 AML patients 65 years or older, were presented at the EHA (*i.e.*, European Hematology Association) meeting in 2014 in Milano [13]. In this study, older AML patients with newly-diagnosed or secondary AML with >30% bone marrow blasts and white blood cell counts ≤15 × 10^9^/L (prior hydroxyurea allowed) were pre-selected to receive one of three regimens per investigator’s choice (*i.e.*, intensive chemotherapy (standard “7 + 3” regimen), low-dose cytarabine (Ara-C) (20 mg twice per day SC for 10 days of each 28-day cycle) or best supportive care only. Patients were then randomized to receive either azacitidine (*n =* 241) (75 mg/m^2^/day for seven days SC of each 28-day cycle) or their preselected treatment (*i.e.*, conventional care regimen (CCR)) (*n =* 247). Median OS, the primary endpoint of the study, was 10.4 months for patients receiving azacitidine compared to 6.5 months for patients receiving CCR, which did not reach statistical significance (HR = 0.84 (95% CI 0.69–1.02), *p =* 0.0829). Primarily patients with poor risk cytogenetics (HR = 0.68 (95% CI 0.5–0.94)) and those with AML with dysplasia (HR = 0.69 (95% CI 0.48–0.98)) benefitted from azacitidine compared with CCR. Additionally, a pre-specified sensitivity analysis for OS that censored patients at the start of subsequent AML therapy was conducted. Results of this analysis showed a longer median OS for patients receiving azacitidine (median: 12.1 months) compared to patients receiving CCR (median: 6.9 months) (stratified HR = 0.76 (95% CI 0.60–0.96), *p =* 0.019). Remarkable was the superior outcome of patients who received azacitidine as the first subsequent therapy after CCR (*n =* 21) compared with those patients who received a cytarabine-based treatment as the first subsequent therapy after azacitidine upfront treatment (*n =* 21) (median OS 8.0 *vs.* 3.6 months (*p =* 0.01)). With the intention to treat analysis, the one-year survival was 47% for patients in the azacitidine arm compared to 34% for patients in the CCR arm.

## 4. Decitabine and AML

Decitabine has been studied in AML patients in various dosing schedules. In a phase 2 study of decitabine, patients over 60 years old with untreated AML (*n =* 227; median age 72 years) and ineligible for induction chemotherapy were treated with decitabine [14]. In this study, patients received decitabine 15 mg/m^2^ IV for three hours every eight hours for three days (total: 135 mg/m^2^), which was repeated every six weeks. A median of two cycles was administered (range, 1–4). Patients who completed four cycles of treatment (*n =* 52) subsequently received a median of five maintenance courses (range, 1–19) with a lower dose of decitabine (20 mg/m^2^) infused over one hour on three consecutive days every 4–6 weeks. The complete and partial remission rate was 26%. Response rates did not differ between patients with or without adverse cytogenetics; patients with monosomal karyotypes also responded. The median overall survival from the start of decitabine treatment was 5.5 months; the one-year survival rate was 28%, and the two-year survival rate was 13%.

In an attempt to find the optimal therapy with decitabine in MDS, Kantarjian and co-workers compared three decitabine schedules administered in an outpatient setting at the Monroe Dunaway Anderson Cancer Center (MDACC): (1) 20 mg/m^2^ intravenously over one hour daily for five days; (2) 20 mg/m^2^ subcutaneously daily, divided into two doses, for five days; or (3) 10 mg/m^2^ intravenously over 1 h daily for 10 days [15]. Courses of decitabine were repeated every four weeks. In the subgroup analysis the, five-day intravenous schedule, which had the highest dose intensity, yielded the highest CR rate (39%) in comparison to the five-day subcutaneous schedule (21%) and the 10-day intravenous (10 mg/m^2^) schedule (24% CR). It should be noted that the 10-day intravenous schedule investigated a dose of 10 mg/m^2^, which is half the dose that was used in the 10-day schedule by Blum *et al.* and Ritchie *et al.* (extensively discussed later) [16,17]. The results of the five-day 20-mg/m^2^ schedule in MDS were confirmed in a larger multi-center study (ADOPT trial, *i.e.*, Alternative Dosing for OutPatient Treatment) [18].

Recently, the results have been reported of a multicenter, randomized, open label, phase 3 trial, which compared the efficacy and safety of decitabine in the five day schedule (20 mg/m^2^, Days 1–5) (*n =* 242) with treatment choice (supportive care (*n =* 28) and low-dose cytarabine (at a dose of 20 mg/m^2^ once daily for 10 days, every four weeks) (*n =* 215)) of older patients with newly-diagnosed AML and poor- or intermediate-risk cytogenetics [19]. Although the planned primary analysis of this trial after 396 deaths did not show a significant improvement of OS with decitabine *vs.* treatment choice (median OS 7.7 months *vs.* 5.0 months), an unplanned analysis after 446 deaths showed a significant benefit for decitabine. The CR rate in this study was 24%. The data from this study led to approval of decitabine for the treatment of AML in Europe, but not in the U.S. (*i.e.*, >30% blasts). The FDA has approved both azacitidine and decitabine for the treatment of all MDS subtypes (up to 30% blasts). The EMA (*i.e.*, European Medicines Agency) has approved azacitidine for high risk MDS, chronic myelomonocytic leukemia (with less than 10% myeloblasts, monoblasts, and promonocytes in bone marrow, *i.e.*, CMML-1) and AML (up to 30% blasts). In Table 1 a summary is provided of clinical outcome of phase 3 trials of azacitidine and decitabine in AML.

## 5. Azacitidine and Decitabine in 10-Day Schedules

Azanucleotides need cell cycling to become incorporated into the DNA during the S phase. Since cell cycling is essential to effect methylation reversal, it could be argued that prolonged administration (e.g., 10 days) of azanucleotides could be pharmacodynamically superior to standard schedule (five days for decitabine and seven days for azacitidine), Table 2 Clinical Outcome dependent on dosing in AML.

**Table 1:** Clinical outcome of phase 3 trials of azacitidine and decitabine in acute myeloid leukemia (AML).

Azacitidine (7 Days 75 mg/m^2^ SC; Every 4 Weeks)				
Study	Competitors	CR (%)	Median OS	1/2-Year OS
Post hoc analysis CALGB 9221 (AML 20%–30% blasts) [11]	AZA (*n =* 27) *vs.* Observation (*n =* 12)	7% *vs.* 0%	19.3 months *vs.* NA; Combining CALGB 8421, 8921, 9221: 12.9 months (*n =* 25; *p =* NA)	NA
Post hoc analysis AZA001 study (AML 20%–30% blasts) [12]	Aza (*n =* 55) *vs.* CCR (*n =* 58) (BSC = 27/LDAC = 20/IC = 11)	18% *vs.* 16%	24.5 months *vs.* 16 months (*p =* 0.005)	50% *vs.* 16% (*p =* 0.001) (2-year OS)
AML001 study (AML >30% blasts) [13]	Aza (*n =* 241) *vs.* CCR (*n =* 247) (BSC = 45/LDAC = 158/IC = 44)	20% *vs.* 22%	10.4 months *vs.* 6.5 months (*p =* 0.08). Analysis censored for subsequent Tx: 12.1 months *vs.* 6.9 months (*p =* 0.01)	46.5% *vs.* 34.2% (*p =* NA) (1-year OS)
**Decitabine (5 Days 20 mg/m^2^ IV; Every 4 Weeks)**				
DACO-016 (AML >20% blasts; only intermediate and poor risk) [19]	Decit (*n =* 242) *vs.* TC (*n =* 243) (BSC = 28/LDAC = 215)	15.7% *vs.* 7.4%	7.7 months *vs.* 5.0 months (*p =* 0.11). Analysis censored for subsequent Tx: 8.5 months *vs.* 5.3 months (*p =* 0.04). Unplanned analysis after 446 deaths: 7.7 months *vs.* 5.0 months (*p =* 0.04)	NA

CR: complete remission; OS: overall survival; CALGB: Cancer and Leukemia Group B; AZA: azacitidine; CCR: conventional care regimen; BSC: Best Supportive Care; LDAC: low dose Ara-C; Tx: treatment; DACO: Dacogen, decitabine; Decit: decitabine; TC: treatment choice.

**Table 2:** Clinical outcome dependent on dosing in AML.

Azacitidine			
Study	Dosing	CR (%)	Median OS
Post hoc analysis AML001 study (phase 3) (AML 20%–30% blasts) [12]	Aza (*n =* 55) 7 days 75 mg/m^2^ SC; every 4 weeks	18%	24.5 months
AML001 study (phase 3) (AML >30% blasts) [13]	Aza (*n =* 241) 7 days 75 mg/m^2^ SC; every 4 weeks	20%	10.4 months
United States Leukemia Intergroup Trial E1905 (phase 2) [20]	Aza (*n =* 74) 10 days 50 mg/m^2^ SC; every 4 weeks	12%	18 months
**Decitabine**			
German phase 2 study [14]	Decit (*n =* 227) 3 days (135 mg/m^2^ total)	13%	5.5 months
DACO-016 (phase 3) (AML >20% blasts; only intermediate and poor risk) [19]	Decit (*n =* 242) 5 days 20 mg/m^2^	15.7%	7.7 months
Ohio State University experience (phase 2) [16]	Decit (*n =* 53) 10 days 20 mg/m^2^	47%	12.7 months
Cornell University experience (report of retrospective analysis) [17]	Decit (*n =* 52) 10 days 20 mg/m^2^	40% (after excluding 6 patients who received prior azanucleotide CR = 46%)	10.5 months

In a recent open label phase 2 randomized trial, azacitidine in a dose of 50 mg/m^2^/day was given for 10 days ± entinostat 4 mg/m^2^/day on Day 3 and Day 10 [20]. One hundred forty nine patients were analyzed, including 97 patients with MDS and 52 patients with AML. In the 10-day azacitidine group, 32% (95% CI, 22% to 44%) experienced hematological normalization (HN) (*i.e.*, complete remission + partial remission + tri-lineage hematological improvement). Although CR rates of the 10-day schedule were comparable with the reported CR rates with the seven-day schedule, this study suggest that prolonged administration of azacitidine seems to increase the HN rate compared with standard dosing (almost doubling of HN compared with the historical control Cancer and Leukemia Group B 9221 trial). Median overall survivals were 18 months for the 10-day azacitidine schedule and 13 months for the group with combined treatment of azacitidine and entinostat.

The experience with decitabine in a 10-day schedule of decitabine (of 20 mg/m^2^) in AML patients has also been reported [16,17]. The data on the 10-day schedule are intriguing. The 10-day schedule was explored in a phase 2 clinical trial (*n =* 53) with single-agent decitabine in older patients (≥60 years) with previously untreated AML, who were not candidates or who refused intensive chemotherapy [16]. Subjects were treated with decitabine 20 mg/m^2^ intravenously over 1 h on Days 1 to 10. Nineteen patients (36%) had antecedent hematologic disorder or therapy-related AML; 16 had complex karyotypes (≥3 abnormalities). The CR rate was 47% (*n =* 25), achieved after a median of three cycles of therapy. Nine additional subjects had no morphologic evidence of disease with incomplete count recovery, for an overall response rate of 64% (*n =* 34). CR was achieved in 52% of subjects presenting with normal karyotype (11 of 21) and in 50% (8 of 16) of those with complex karyotypes (defined as ≥3 abnormalities). Death occurred within eight weeks in 15% of subjects. The CR rate in subjects presenting WBC counts ≥15 × 10^9^/L (range, 15 × 10^9^/L–150 × 10^9^/L) was 57% (eight of 14 subjects), including 50% (four of eight) for those subjects presenting WBC count ≥50 × 10^9^/L. The disease-free survival duration was 46 weeks. The median OS was 55 weeks. Furthermore, this study showed that decitabine was well tolerated. Although patients were neutropenic for prolonged times, they did not experience mucositis and, therefore, could be managed largely in the outpatient setting. Patients who had less than 5% bone marrow blasts around Day + 28 of the 10-day decitabine cycle continued with a five-day schedule. Those patients with more than 5% bone marrow blasts received another 10-day decitabine cycle.

Recently, the efficacy and safety data of another study exploring the 10-day decitabine schedule have been reported [17]. In this study, 52 newly-diagnosed, older AML patients were treated with the 10-day decitabine schedule. All patients received at least one 10-day induction cycle with decitabine 20 mg/m^2^ intravenously. After CR, most of the patients were treated with ongoing five-day cycles of decitabine 20 mg/m^2^ until toxicity or progression of disease. The median number of treatment cycles was two (range 1–18). Patients required a median number of two cycles (range, 1–4) to achieve a response, with a median time to CR of 55.5 days (range 18–122 days). Among the 52 patients, 21 (40%) achieved a CR. None of the six patients who had previously been treated with azacitidine or decitabine for MDS achieved a CR. If these patients are excluded from the study population, the CR rate becomes 46%. Responses were durable over one year. The median OS was 318 days. The extra medullary toxicity was mild, but myelosuppression was noted in all patients. The median time to neutrophil recovery was 42.5 days (range, 20–120 days), and the median time to platelet count recovery was 48 days (range, 1–130 days). Twenty-nine patients (55%) had neutropenic fever with bacteremia requiring intravenous antibiotics. Patients were hospitalized for a median of 39 days (range, 0–169 days). However, it should be noted that the authors state in the manuscript that the reasons for prolonged hospitalization were frequently social or logistical, rather than medical.

These single-center experiences of the 10-day schedule of decitabine show promising remission and survival results. The 20 mg/m^2^ daily decitabine for five-day or 10-day schedules are currently compared in a prospective randomized study in older unfit AML patients at the MD Anderson Cancer Center (NCT01786343, see ClinicalTrials.gov). The reported CR rates achieved with the 10-day decitabine schedule are comparable with those achieved after intensive chemotherapy (e.g., HOVON43 (a study of the Dutch HOVON foundation, *i.e.*, Hemato-oncologie voor Volwassenen Nederland) reported a CR rate of 54% after conventional intensive chemotherapy in AML patients ≥60 years) [21]. These CR rates should be considered with the perspective that hypomethylating agents impact survival without inducing CR, suggesting that almost similar CR rates between 10-day decitabine and conventional chemotherapy might translate to a survival benefit for the 10-day decitabine schedule. Therefore, the 10-day decitabine schedule might provide a framework upon which to build future combination studies to improve outcomes for older AML patients. The European Organisation for Research and Treatment of Cancer (EORTC) Leukemia Group, together with the Gruppo Italiano Malattie EMatologiche dell’Adulta (GIMEMA), the Central European Leukemia Group (CELG) and the German MDS Study Group, recently opened a prospective randomized trial to compare conventional intensive chemotherapy based on cytarabine combined with an anthracycline (“3 + 7”) with the hypomethylating agent, decitabine, to determine the optimal backbone for the treatment of older AML patients (NCT02172872).

## 6. Hypomethylating Agents as Maintenance Therapy

Because of its low toxicity profile, maintenance treatment with hypomethylating agents is an attractive treatment option for older AML patients who are at high risk for relapse and who are not candidates for allogeneic hematopoietic cell transplantation. A prospective phase 2 study demonstrated that maintenance azacitidine treatment for five days at a dose of 60 mg/m^2^ (after previous treatment with intensive chemotherapy) is safe and feasible [22]. Although no positive effect on the duration of CR was observed, this limited efficacy should be considered with the perspective of the low number of patients (*n =* 23, including 10 patients with MDS/AML) included. Currently, the HOVON is performing a prospective randomized trial comparing maintenance treatment with azacitidine (five days at a dose of 50 mg/m^2^) with observation in older AML patients who are in CR after at least two cycles of intensive chemotherapy. The efficacy of oral azacitidine in the maintenance setting, which is logistically very attractive, is currently being tested in a prospective randomized study (Quazar AML-001; NCT01757535).

Limited data are available on the efficacy of decitabine in the maintenance setting. Lübbert *et al.* have reported three-day decitabine maintenance (20 mg/m^2^/day) in 43 older AML patients who had received four cycles of decitabine treatment [14]. Further improvement of treatment response on maintenance or relapse after complete remission was not systematically recorded. Recently, the results of a small prospective phase 3 trial were reported. In this study, maintenance treatment with decitabine (*n =* 20) was compared with conventional care (*n =* 25; six observation, nine low-dose cytarabine and 10 intensive chemotherapy) [23]. Baseline characteristics were relatively balanced, with patients in the decitabine arm being older (55% >60 years *vs.* 28% in the conventional care arm and all with intensive chemotherapy being <60 years) and somewhat more frequently having poor karyotypes (25% *vs.* 20%). Decitabine was administered as 20 mg/m^2^ IV daily on day 1–5 every 4–8 weeks for a total of up to 12 cycles. The primary endpoint was the relapse rate at one year. After a median follow-up of 44.9 months, fewer patients in the decitabine arm relapsed (50% *vs.* 60%), and the OS rate was 45% in the decitabine *vs.* 36% in the conventional care group (HR = 0.63; *p =* 0.32); these differences were not statistically significant. Treatment with standard-dose decitabine was well tolerated, with the most common adverse events being uncomplicated grade 3 neutropenia and/or thrombocytopenia.

## 7. DNA Hypomethylating Agent Therapy as a Bridging Strategy to AlloHCT

Various studies, as discussed above, have strongly suggested that the DNA hypomethylating agents, azacitidine and decitabine, have a favorable outcome in older AML patients. However, improvement is relatively small when compared to life expectancy in the absence of disease (in the Netherlands, a 76-year-old person has a life expectancy of 11 years), and treatment with hypomethylating agents cannot be considered curative. Treatment with hypomethylating agents could be curative when used in a sequential approach: debulking the disease with hypomethylating agents followed by reduced toxicity conditioning and allografting. In this strategy, hypomethylating agents are used as a bridging strategy before allografting; an effective, but non-toxic drug, like azacitidine or decitabine, is used to allow also more fragile patients to reach the potential curative treatment of allografting [24]. Azanucleotide treatment aims at debulking the disease while a donor (most often, an unrelated donor) is identified. Notably, because non-hematologic toxicities are usually mild and manageable (decitabine does not result in mucositis), the patient has the chance to stay fit and ambulatory, and the performance status may be improved with a good quality of life. This approach is by now well established in the literature (more than 120 patients have been reported up to now) as both feasible and capable of inducing longer-term remissions without negative effects with respect to graft-*versus*-host disease [25,26,27,28,29,30].

The impact of prior-to-transplantation azacitidine treatment on patient outcome after allogeneic hematopoietic cell transplantation (alloHCT) in MDS has been reported by a large French study [31]. In this study, 265 consecutive patients underwent alloHCT for MDS between October, 2005, and December, 2009; 163 had received cytoreductive treatment prior to transplantation, including induction chemotherapy (ICT) alone (ICT group; *n* = 98), azacitidine alone (AZA group; *n* = 48), or azacitidine preceded or followed by ICT (AZA-ICT group; *n* = 17). At diagnosis, 126 patients (77%) had an excess of marrow blasts and 95 patients (58%) had intermediate-2 or high-risk MDS according to the International Prognostic Scoring System (IPSS). Progression to more advanced disease before alloHCT was recorded in 67 patients. Donors were siblings (*n* = 75) or HLA (Human Leucocyte Antigen)-matched unrelated (10/10; *n* = 88). They received blood (*n* = 142) or marrow (*n* = 21) grafts following either myeloablative (*n* = 33) or reduced intensity (*n* = 130) conditioning. With a median follow-up of 38.7 months, three-year outcomes in the AZA, ICT and AZA-ICT groups were: 55%, 48% and 32% (*p* = 0.07) for OS; 42%, 44% and 29% (*p* = 0.14) for event-free survival (EFS); 40%, 37% and 36% (*p* = 0.86) for relapse; and 19%, 20% and 35% (*p* = 0.24) for non-relapse mortality (NRM), respectively. Multivariate analysis confirmed the absence of statistical differences between the AZA and the ICT groups in terms of OS, EFS, relapse and NRM.

The value of alloHCT after hypomethylating therapy was studied in a German study [32]. The multivariate analysis of this study, to minimize selection bias, was limited to patients aged 60–70 years with high-risk *de novo* MDS or secondary AML, who either received alloHCT (*n =* 105; with at least intermediate intensity conditioning in about half of the cases after induction chemotherapy) or who received azacitidine, but not alloHCT (*n =* 75), because of a lack of a donor or institutional guidelines. After accounting for performance status, cytogenetics, time from diagnosis and blast%, alloHCT was associated with superior OS compared to azacitidine without alloHCT, with the difference in OS becoming apparent one year after initiation of treatment (two-year OS 39 *vs.* 23%).

## 8. Use of Hypomethylating Agents after AlloHCT

Demethylating agents have been used after alloHCT in different settings: (1) to maintain CR (*i.e.*, to prevent relapse); (2) preemptive; and (3) to treat relapse. Limited data are available for whether maintenance therapy with HMAs after alloHCT improves RFS. In a dose finding study in high risk MDS/AML patients who were in CR at Day + 30 after transplantation, azacitidine was given subcutaneously for five subsequent days, starting on the sixth week after alloHCT at one of five dose levels (8, 16, 24, 32, 40 mg/m^2^) [33]. The dose of 32 mg/m^2^ was chosen. Azacitidine did not affect engraftment. At a median follow-up of 20.5 months, the one-year EFS and OS were 58% and 77%, respectively. Because most acute graft-versus-host disease (GVHD) started before starting azacitidine and patients with severe GVHD were excluded, no firm conclusions regarding acute GVHD could be made. The probability of developing chronic GVHD was, however, decreased significantly with longer azacitidine treatments. Currently, the maintenance question with HMAs after alloHCT is being evaluated in several clinical trials (NCT01168219, NCT01995578 and NCT01541280).

The value of azacitidine as a minimal residual disease-based preemptive therapy after alloHCT has been reported in a cohort of 59 patients who were prospectively monitored for impending relapse by decreasing CD34^+^ cell chimerism [34]. In this trial (RELAZA, Azacitidine for treatment of imminent relapse in MDS or AML patients after alloHCT), at a median of 169 days after alloHCT, 20/59 prospectively screened patients experienced a decrease of CD34^+^ donor chimerism to <80% and received four azacitidine cycles (75 mg/m^2^/day for seven days) while in complete hematologic remission. A total of 16 patients (80%) responded with either increasing CD34^+^ donor chimerism to ≥80% (*n =* 10; 50%) or stabilization (*n =* 6; 30%) in the absence of relapse. Hematologic relapse occurred ultimately in 13 patients.

HMAs have been used to treat recurrent disease after alloHCT, and the achievement of remission and complete donor chimerism has been reported [35,36]. Although response rates of around 50% and CR rates around 15% have been reported, the survival rate was still low and comparable with second alloHCT in this setting. The combination of azacitidine and donor lymphocyte infusions (DLI) as the first salvage therapy for relapse after alloHCT has also been studied in a cohort of 30 patients with AML/MDS within a prospective single-arm phase 2 trial [37]. The overall response rate was 30%, and 5/30 patients achieved long-term CR. Acute and chronic GVHD were seen in 37% and 17% of patients, respectively. This study suggests that azacitidine in combination with donor lymphocytes is an active treatment in high-risk patients who have relapsed after alloHCT.

## 9. Treatment of Older AML Patients

The optimal treatment of older AML patients in daily clinical practice remains challenging and is dependent on patient characteristics (age, co-morbidity), disease characteristics (cytogenetic and molecular abnormalities, WBC, *etc.*) and the wishes of the patient.

The OS of older AML patients has not been improved during the last few decades with intensive chemotherapy based on cytarabine combined with an anthracycline [38]. Although older AML patients generally have a limited benefit with currently available treatment, only a few prospective randomized studies in older AML patients, comparing different treatment strategies, have been done. A prospective clinical trial, though with a limited number of patients (*n =* 60), reported that standard intensive treatment improves early death rates and long-term survival compared to the best supportive care [39], a finding that was confirmed by an analysis of the Swedish Acute Leukemia Registry [40]. A prospective study in older patients with *de novo* AML (*n =* 87) has compared intensive chemotherapy with low-dose cytarabine (20 mg/m^2^ for 21 days) and reported a similar overall survival (OS) in both arms, despite a higher number of complete remissions (CRs) in the intensive chemotherapy arm [41]. Moreover, a prospective randomized trial (*n =* 202) demonstrated that low-dose cytarabine (20 mg twice daily for 10 days) treatment was superior to the best supportive care and hydroxyurea [42]. In this study, patients with adverse cytogenetic profiles did not benefit from low-dose cytarabine. These studies suggest that older AML patients benefit from treatment, either by intensive chemotherapy or by low-dose cytarabine. From the perspective that the superiority of intensive chemotherapy (*i.e.*, “3 + 7”) over less intensive therapy (e.g., low-dose Ara-C) has not been conclusive, the recently reported study, comparing decitabine, administered as a five-day regimen, with low-dose Ara-C, in older AML patients (*n =* 485), is particularly interesting [19]. Indeed, this study showed, though at an unplanned analysis with a one-year extended follow-up for survival, that the five-day decitabine treatment resulted in a superior overall survival compared with the low-dose Ara-C treatment.

No published data of prospective randomized trials that have compared the efficacy of intensive chemotherapy (“3 + 7”) with hypomethylating agents are currently available. The MD Andersen Cancer Center reported the results of a cohort study of 671 patients, including 114 patients treated with hypomethylation-based (either azacitidine or decitabine) therapy and 557 patients treated with intensive chemotherapy [43]. Both groups were balanced according to cytogenetics and performance status and were older than 65 years. The CR rates with chemotherapy and hypomethylating agents were 42% and 28%, respectively (*p =* 0.001), and the eight-week mortality 18% and 11%, respectively (*p =* 0.075). Two-year relapse-free survival rates were 28% (chemo) *vs.* 39% (DNA methyltransferase inhibitor), *p =* 0.84. The median OS (6.7 *vs.* 6.5 months, *p =* 0.41) were similar in the two groups. Multivariate analysis confirmed that the type of AML therapy (intensive chemotherapy or hypomethylating agents) was not an independent prognostic factor for survival. Interestingly, in this study, multivariate analysis revealed that decitabine was associated with improved median OS compared with azacitidine (8.8 *vs.* 5.5 months, respectively, *p =* 0.03). This is in line with our own published experience in 200 consecutive older AML patients [44,45]. It should be noted that the observations of this retrospective analysis also suggest that the currently used response criteria (*i.e.*, CR) are not sufficient for evaluating some (less intensive) treatment strategies.

## 10. Conclusions

In conclusion, treatment recommendations for older adults with AML need to be individualized based on disease and patient characteristics [46]. However, at this moment, available clinical trial data do not satisfactorily determine which older patients are likely to benefit from specific treatments, given the complexity of tumor and patient characteristics underlying treatment responsiveness and treatment tolerance. The question to be answered is: what is considered a favorable treatment outcome that justifies a certain treatment-related mortality? From this perspective, it should be noted that a hematopoietic cell transplantation comorbidity index (HCT-CI) ≥3 has been reported to be associated with 29% mortality within 28 days from time of intensive chemotherapy in a cohort of patients, 177 AML patients over 60 years of age [47]. Should we treat these patients (with HCT-CI ≥3) with hypomethylating agents? Should the genotype of the AML blasts influence this decision? Without a doubt, azacitidine and decitabine are valuable treatment options for older AML patients, especially patients with co-morbidities and intermediate and poor risk disease. Whether azanucleotide treatment is superior (or comparable) with intensive chemotherapy, especially when used as a bridge to allogeneic transplantation, is an open question. To determine the optimal relation between certain treatments and disease (e.g., genotype) and patient-related factors (e.g., co-morbidity), future studies in older AML patients should include extensive biomarker analyses and geriatric assessments.

Cytarabine, azacitidine and decitabine are cytidine analogs. Cytarabine combines a cytosine base with an arabinose sugar. The carbon-5 of the cytidine backbone is substituted by nitrogen in the azanucleotides (azacitidine and decitabine). Azacitidine is intracellularly converted to 5-aza-2′-deoxycytidine (decitabine) by ribonucleotide reductase. Decitabine is incorporated in place of cytidine into DNA, where it acts as a direct and irreversible inhibitor of DNMTs. Because of the nitrogen atom at position 5, the enzyme DNMT remains covalently bound to DNA, and its DNA methyltransferase function is blocked and results in the degradation of trapped DNA methyltransferases. Consequently, cells then replicate in the absence of DNMTs, which results in progressive loss of methylation marks and reactivation of previously silenced genes. Little is known about the impact of azacitidine incorporated into RNA.
